# Detection of barriers to dispersal is masked by long lifespans and large population sizes

**DOI:** 10.1002/ece3.3470

**Published:** 2017-10-16

**Authors:** Jordan R. Hoffman, Janna R. Willoughby, Bradley J. Swanson, Kevin L. Pangle, David T. Zanatta

**Affiliations:** ^1^ Biology Department Institute for Great Lakes Research Central Michigan University Mount Pleasant MI USA; ^2^ Department of Biological Sciences Purdue University West Lafayette IN USA

**Keywords:** effective population size, fragmentation, generation time, microsatellites, *Quadrula quadrula*, simulation

## Abstract

Population genetic analyses of species inhabiting fragmented landscapes are essential tools for conservation. Occasionally, analyses of fragmented populations find no evidence of isolation, even though a barrier to dispersal is apparent. In some cases, not enough time may have passed to observe divergence due to genetic drift, a problem particularly relevant for long‐lived species with overlapping generations. Failing to consider this quality during population structure analyses could result in incorrect conclusions about the impact of fragmentation on the species. We designed a model to explore how lifespan and population size influence perceived population structure of isolated populations over time. This iterative model tracked how simulated populations of variable lifespan and population size were affected by drift alone, using a freshwater mussel, *Quadrula quadrula* (mapleleaf), as a model system. In addition to exhibiting dramatic lifespan variability among species, mussels are also highly imperiled and exhibit fragmentation by dams throughout the range of many species. Results indicated that, unless population size was small (<50 individuals) or lifespan short (<22 years), observing genetic divergence among populations was unlikely. Even if wild populations are isolated, observing population structure in long‐lived mussels from modern damming practices is unlikely because it takes longer for population structure to develop in these species than most North American dams have existed. Larger population sizes and longer lifespans increase the time needed for significant divergence to occur. This study helps illuminate the factors that influence genetic responses by populations to isolation and provides a useful model for conservation‐oriented research.

## 1 INTRODUCTION

Population genetic analyses have become invaluable assessment tools for conservation of species inhabiting fragmented landscapes. Habitat fragmentation splits populations across habitat patches, decreasing or even halting dispersal and gene flow (Couvet, 2002). Fragmented populations that remain isolated for several generations tend to diverge genetically over time, causing the formation of population genetic differentiation and structure between fragments (Slatkin, 1987). This population genetic differentiation is largely due to the evolutionary force known as genetic drift.

The random effects of genetic drift reduce variation within a population and, while not necessarily deleterious, often result in negative impacts (reviewed in Keller & Waller, 2002). Small or isolated populations will experience genetic drift to some degree, typically resulting in an increase in homozygosity. While the resulting increase in inbreeding may lead to inbreeding depression (e.g., O'Grady et al., 2006; Pekkala, Knott, Kotiaho, Nissinen, & Puurtinen, 2014), this is not always the case (e.g., Hedrick et al. 2016; Quaglietti et al., 2017; Smith, 1995). Additionally, given enough time, other forces can ameliorate the impact of increased homozygosity including genetic purging (Boakes, Wang, & Amos, 2007; Glémin, 2003; Lopez‐Cortegano, Vilas, Caballero, & Garcia‐Dorado, 2016), increased gene flow (Garcia‐Navas et al., 2015) or selection that can overwhelm the force of genetic drift (Bouzat, 2010). However, when inbreeding depression is not alleviated, effects such as decreased reproduction can lead to population crashes (Liberg et al., 2005; Räikkönen, Vucetich, Peterson, & Nelson, 2009). Thus, the potential for inbreeding depression in small and isolated populations resulting from genetic drift prompts analyses of genetic structure among populations to study population fragmentation and the erection of barriers to gene flow.

In some instances, analyses of fragmented populations find no evidence of fragmentation or isolation, even though a barrier to dispersal seems apparent. Occasionally, the dispersal ability of the species in question may have been underestimated, or patches may not be as isolated as expected. Reddy et al. (2012), for example, identified little population structure in tigers (*Panthera tigris*) between two increasingly fragmented preserves. However, low levels of structure between nearby fragments were explained by the high dispersal tendencies of subadult tigers seeking to establish new territories (Reddy et al., 2012). In other cases, failure to observe genetic structure may be due to molecular marker choice that provides little power for observing the effects of isolation.

In some situations, low or no observed structure between clearly isolated populations may be explained by other factors. It is often possible that insufficient time has elapsed to successfully identify divergence of isolated populations due to genetic drift. This may be a result of long generation time increasing the time it takes for a population to respond genetically to isolation. For instance, Wozney, Haxton, Kjartanson, and Wilson (2011) found no structure in lake sturgeon (*Acipenser fulvescens*) across waterways fragmented by dams for nearly 100 years. Similarly, Marsack and Swanson (2009) observed little genetic differentiation resulting from habitat fragmentation in eastern box turtles (*Terrapene carolina carolina*). In both cases, the species were long‐lived and had strongly overlapping generations, which results in ecological processes, such as fragmentation, occurring at a faster rate than evolutionary processes.

In addition to generation time, genetic response time to isolation is influenced by a population's effective size (*N*_e_). Introduced by Wright (1931), *N*_e_ is the size of an idealized population size that loses genetic diversity at the same rate as the focal population, with respect to drift. In natural populations, a variety of factors such as unequal sex ratios and temporally variable population size reduces *N*_e_ relative to the census size (*N*_c_; Frankham, 2007). Furthermore, populations with small *N*_e_ will lose genetic variation faster than those with larger *N*_e_ (Allendorf, 1986). Because population fragmentation breaks populations into subsets and generally decreases *N*_e_, fragmentation typically results in populations that are more susceptible to the influence of drift. Populations, particularly those with smaller *N*_e_, require immigration or gene flow from outside sources in order to negate the influence of drift. However, populations with a large *N*_e_, even after fragmentation, are slow to change due to drift, increasing the lag in detectable changes to population genetics. As such, the *N*_e_ of fragmented populations will also influence the results in structure analyses in conjunction with the long generation time of the organism. Failure to account for long generation times or large population sizes during a genetic structure analysis could cause an inaccurate interpretation of results; sampling a population too early after an isolation event could reveal little or no structure, leading to the inappropriate conclusion that gene flow still occurs between fragments. Thus, the ability to understand and predict the genetic response lag of a population, in years, to connectivity changes based on its lifespan and population size would be a valuable resource for researchers and conservation managers.

Manipulative models and simulations can be used to explore the relationships between multiple variables and generate predictions. Landguth et al. (2010), for example, used a simulative approach to detect gene flow barriers of variable intensities using the response lag of populations to fragmentation. Other models have been used to examine how landscape conditions and gene flow influence genetic structure in eastern Massasauga rattlesnakes (*Sistrurus* c. *catenatus*; DiLeo, Rouse, Dávila, & Lougheed, 2013) and American pikas (*Ochotona princeps*; Castillo, Epps, Davis, & Cushman, 2014). However, models to date have focused on fragmentation in more short‐lived species, and operate on the order of generations, rather than years. Models that track a population over years, rather than generations, can show slow change occurring within generations, as opposed to just across them. Particularly in long‐lived species, understanding how fragmentation impacts populations on a yearly scale may be more applicable than generations for conservation and management.

The purpose of this study is to use a modeling approach to evaluate the influence of lifespan and population size on the ability to detect population genetic structure. Herein, we detail an iterative population model to examine the influence of lifespan and population size on response lag to fragmentation and isolation as manifested in population structure. To explore response lag with this model, we use a model system of mapleleaf mussels (*Quadrula quadrula*), a particularly long‐lived species of freshwater mussel, sampled from populations potentially isolated by a low‐head (run‐of‐river) dam. In this case study, our model determined if observable genetic divergence should be expected at this time, and if not, when divergence would be observed with 95% probability. Freshwater mussels vary among species in lifespan by an order of magnitude, making them particularly useful model organisms to assess the influence of lifespan on the lag in response to fragmentation.

## 2 MATERIALS AND METHODS

### 2.1 Case study

**Figure 1:**
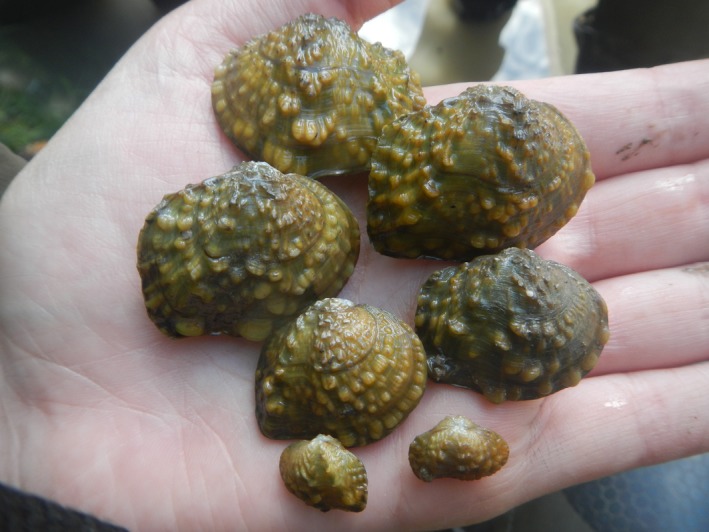
Mapleleaf mussel (*Quadrula quadrula*)

*Quadrula quadrula* is a freshwater mussel native to the Mississippi‐Ohio river drainage and the central Great Lakes (Figure 1). While not considered an imperiled species in the United States, the species is currently categorized as threatened in Ontario, Canada (Committee on the Status of Endangered Wildlife in Canada (COSEWIC), 2006; Species at Risk Act (SARA), 2016). The lifespan of *Q. quadrula* is typically long, with individuals living 22 years on average and as long as 65 years (Committee on the Status of Endangered Wildlife in Canada (COSEWIC), 2006). In the Grand River of Ontario, *Q. quadrula* inhabit a stretch of river split by the Dunnville Dam, a low‐head dam installed in 1829 on the lower section of the river (Committee on the Status of Endangered Wildlife in Canada (COSEWIC), 2006; MacDougall, Wilson, Richardson, Lavender, & Ryan, 2007). Like other unionid mussels, *Q. quadrula* parasitize the gills of fish during their larval stage of development, using the host as a source of nutrition and mode of dispersal (Haag, 2012). The host fish most likely used by *Q. quadrula* within this region is the channel catfish (*Ictalurus punctatus*, Committee on the Status of Endangered Wildlife in Canada (COSEWIC), 2006). While the channel catfish is capable of long‐distance dispersal (Steward & Watkinson, 2004), the species is incapable of dispersing upstream across low‐head dams (Butler & Wahl, 2011; Welker, 1967). Thus, the Dunnville Dam should act as a barrier to upstream dispersal and gene flow for *Q. quadrula*, isolating upstream mussels from the larger, downstream population.

**Figure 2:**
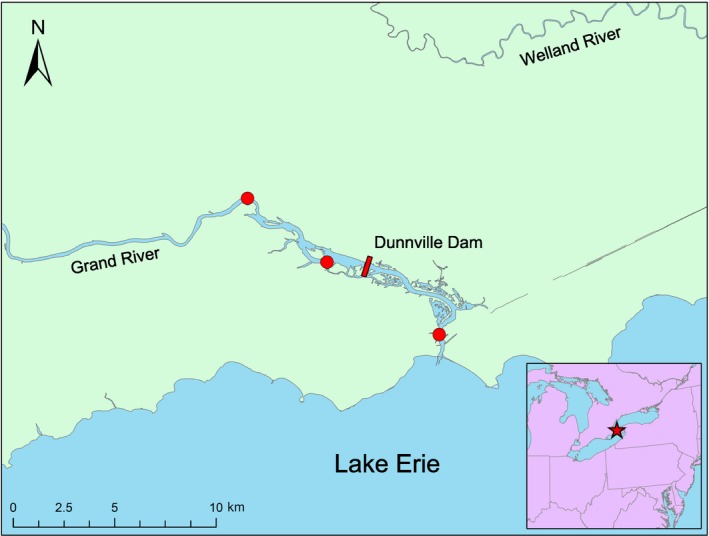
Sampling locations for *Quadrula quadrula* in the lower Grand River, Ontario, Canada. The location of the Dunnville Dam is noted by a red bar

Samples used in this study were collected as part of a population genetic study of Lake Erie and Lake Ontario populations of *Q. quadrula* (Hoffman, 2016). Mantle tissue biopsies were collected nonlethally (Berg, Haag, Guttman, & Sickel, 1995) from *Q. quadrula* at two sites above the Dunnville Dam and one site below in 2008 and 2014, respectively (Figure 2). For each sample, DNA was extracted using a modified alcohol extraction method similar to Sambrook, Fritsch, and Maniatas (1989). Six microsatellite loci were then amplified for *Q. quadrula* samples (C4, C114, A112, A130, R9, and D102) using polymerase chain reaction (PCR) and primers designed by Hemmingsen, Roe, and Serb (2009) for a close relative, the Winged Mapleleaf (*Q. fragosa*), and optimized for *Q. quadrula* (Paterson, Griffith, Krebs, Burlakova, & Zanatta, 2015). Each PCR reaction consisted of 1.0 μa of a DNA sample (extracted DNA diluted 1:10 in nanopure water) and 9.0 μw of PCR cocktail [1× Taq buffer, bovine serum albumin (BSA), deoxyribonucleotide phosphate (dNTP), forward and reverse primers, MgCl_2_ and Taq DNA polymerase, as in Paterson et al. (2015)]. Amplification was conducted using an Eppendorf Mastercycler (Eppendorf, Hamburg, Germany) with locus‐specific settings and visually verified using electrophoresis on 1.5% agarose gel. Amplified microsatellite products were scored and sized at the Natural Resources DNA Profiling and Forensic Centre at Trent University using an Applied Biosystems 3730 Series DNA Analyzer (Applied Biosystems, Foster City, CA). GENEMARKER (SoftGenetics LLC, State College, PA) software was used to score alleles into size classes.

Following amplification and genotyping of microsatellite loci, all loci were tested for null alleles using the program MICROCHECKER v. 2.2.3 (van Oosterhout, Hutchinson, Wills, & Shipley, 2004). Loci grouped by collection locality were tested for linkage disequilibrium using GENEPOP 4.2 (Raymond & Rousset, 1995), and for deviations from Hardy–Weinberg Equilibrium using GENALEX v.6.5 (Peakall & Smouse, 2012). To determine if the Dunnville Dam acted as a barrier, we compared populations of *Q. quadrula* above and below the dam using the program STRUCTURE v.2.3.3 (Pritchard, Stephens, & Donnelly, 2000). STRUCTURE analysis was conducted using 100,000 MCMC, 100,000 burn‐in, and five iterations for each possible number of populations tested (*K*), assuming admixture and correlated allele frequencies. Maximum *K* was equal to the number of sites plus one. Mean log likelihood (mean *LnPK*) values derived using methods described by Evanno, Regnaut, and Goudet (2005) and calculated in STRUCTURE HARVESTER v.0.6.93 (Earl & vonHoldt, 2012) were used to determine the most likely number of populations across the dam. The *F*_ST_ value between the upstream and downstream populations was calculated using GENALEX with 9,999 permutations. A discriminant analysis of principal components (DAPC) was conducted using the R package “Adegenet” (Jombart et al., 2016), and the proportion of individuals in the upstream population assigned to the downstream population was reported.

### 2.2 Population genetic model design

We designed an agent‐based, forward time model to simulate the effects of isolation at multiple population sizes on our ability to detect population structuring (available at https://github.com/jwillou/Isolation-Drift). We began each iteration using allele frequencies from *Q. quadrula* from sites downstream of the Dunnville Dam, observed in Hoffman (2016), to randomly generate the genotypes of individuals in a large population, representing the contiguous Lake Erie population prior to any dam construction and potential fragmentation (*N*_c_ = 1,000). This population, encompassing Lake Erie and its tributaries, is likely so large that it has collectively changed little due to drift over the last 200 years, and as such, likely reflects allele frequencies of Grand River *Q. quadrula* prior to the construction of the dam. The simulated source (Lake Erie) population was allowed to persist for 75 years, slightly more than the longest recorded lifespan for *Q. quadrula* (65 years, Committee on the Status of Endangered Wildlife in Canada (COSEWIC), 2006), in order to ensure all populations were in Hardy–Weinberg Equilibrium at the start of the fragmentation simulation. After 75 years, we simulated an isolation event by randomly sampling a subset of individuals (parameter was varied within simulations to 50, 100, 200, 350, or 500) to create an isolated, upriver population. In both the source and isolated populations, we assumed random mating and Mendelian inheritance of alleles, and removed individuals from the population with a probability equal to [1—the annual survival rate] (estimated from the closely related species from tribe Quadrulini, *Amphinaias pustulosa*, 96% annually, Haag, 2012) or when the maximum age was reached (varied from 2 to 102 years using 20‐year intervals). Maximum age was determined to be the maximum recorded lifespan—the estimated age of reproductive maturity (3 years for *Q. quadrula*, Haag, 2012), and individuals entering the population were assumed to have reached reproductive maturity.

We ran each of the 100 replicates of each parameter set out to 400 years (after the 75‐year stabilization of the source population). At the end of the simulation, we estimated average heterozygosity at each year and used STRUCTURE, discriminant analysis of principal components (DAPC), and *F*_ST_ estimates to determine if the imposed isolation was detectable. For STRUCTURE analyses within each iteration, we compared the starting population (year = 0) with the population at 25‐year intervals (between time 25 and 400). DAPC and *F*_ST_ analyses were performed on a yearly basis, rather than on 25‐year intervals. A randomly selected subset of 50 individuals per population (source and isolated) for each analysis was used to maintain consistent sample sizes, regardless of the simulated population size. As in the case‐study analysis, we used a standardized set of parameters (*K* = 1–3; MCMC = 100,000; burn‐in = 100,000; iterations = 5) for each analysis. Structure analyses were facilitated within R using the package “strataG” (Archer, 2014). We determined the most likely *K* by comparing mean *LnPK* within each replicate. For each tested *N*_c_, the frequency of identification of two populations, or the frequency of identifying genetic divergence upstream and downstream of the dam, was calculated. DAPC and *F*_ST_ estimations were performed in the model using the packages “Adegenet” (Jombart et al., 2016) and “DiveRsity” (Keenan, 2017). We interpreted the DAPC results by averaging the proportion of individuals in the isolated population that assigned to the source population cluster across lifespan and population size replicates. Similarly, we averaged the *F*_ST_ estimates between the source and isolated populations across replicates. In all we ran 30 unique parameter sets or a total of 3,000 simulations; this included 20 STRUTURE analyses per replicate (total of 60,000 STRUCTURE runs) and 400 DAPC and *F*_ST_ estimates per replicate (total of 1.2 million estimates for each statistic). All simulations were executed in R version 3.2 (R Development Core Team, 2013) on a high‐performance computing cluster where each node had 20 cores each (dual 10‐core Intel(R) Xeon(R) E5‐2660 v3 CPU), with 256 GB memory per node.

## 3 RESULTS

### 3.1 Case study

In total, 63 *Q. quadrula* samples from the two sites above the Dunnville Dam and 27 samples from the site below the dam were successfully amplified for at least four of six microsatellite loci. Eighty percent of samples were successfully amplified at all loci, while only 5% were amplified at just four loci. One locus, R9, was fixed at all three sampling sites, although greater diversity was observed at other Lake Erie sites (Hoffman, 2016). Potential null alleles were predicted at low frequencies for all six loci (0.09%–7.45%). However, simulations suggest that null alleles at a frequency <20% have little effect on population‐based analyses (Carlsson, 2008; Dakin & Avise, 2004). Following Bonferroni correction (α = 0.0006), no significant deviations from Hardy–Weinberg Equilibrium (HWE) were detected at any loci from any of the three sampling locations (Table 1). No linkage between loci was observed after Bonferroni correction (α = 0.003). STRUCTURE identified only a single population among Grand River sites (*K *=* *1, mean *LnPK* = −1079.96). The *F*_ST_ value between the upstream and downstream populations was 0.047. DAPC assigned 9.98% of individuals from the upstream population to the downstream population.

**Table 1:** Number of *Quadrula quadrula* genotyped (*N*), observed heterozygosity (*H*_o_), and expected heterozygosity (*H*_e_) by sampling site

	LG	GRA1	GRA2
Overall			
* N*	27	43	20
* H*_o_	0.441	0.508	0.539
* H*_e_	0.452	0.478	0.511
A112			
* N*	19	43	19
* H*_o_	0.895	0.791	0.947
* H*_e_	0.69	0.677	0.741
A130			
* N*	17	43	20
* H*_o_	0.588	0.837	0.85
* H*_e_	0.804	0.785	0.665
C4			
* N*	27	43	19
* H*_o_	0.704	0.744	0.684
* H*_e_	0.727	0.804	0.785
C114			
* N*	26	43	20
* H*_o_	0.462	0.651	0.6
* H*_e_	0.489	0.58	0.649
D102			
* N*	27	43	20
* H*_o_	0	0.023	0.15
* H*_e_	0	0.023	0.224
R9			
* N*	25	43	20
* H*_o_	0	0	0
* H*_e_	0	0	0

*N*,* H*_o_, and *H*_e_ are also listed per locus per site LG is located downstream of the Dunnville Dam, while GRA1 and GRA2 are upstream.

### 3.2 Population genetic model

**Figure 3:**
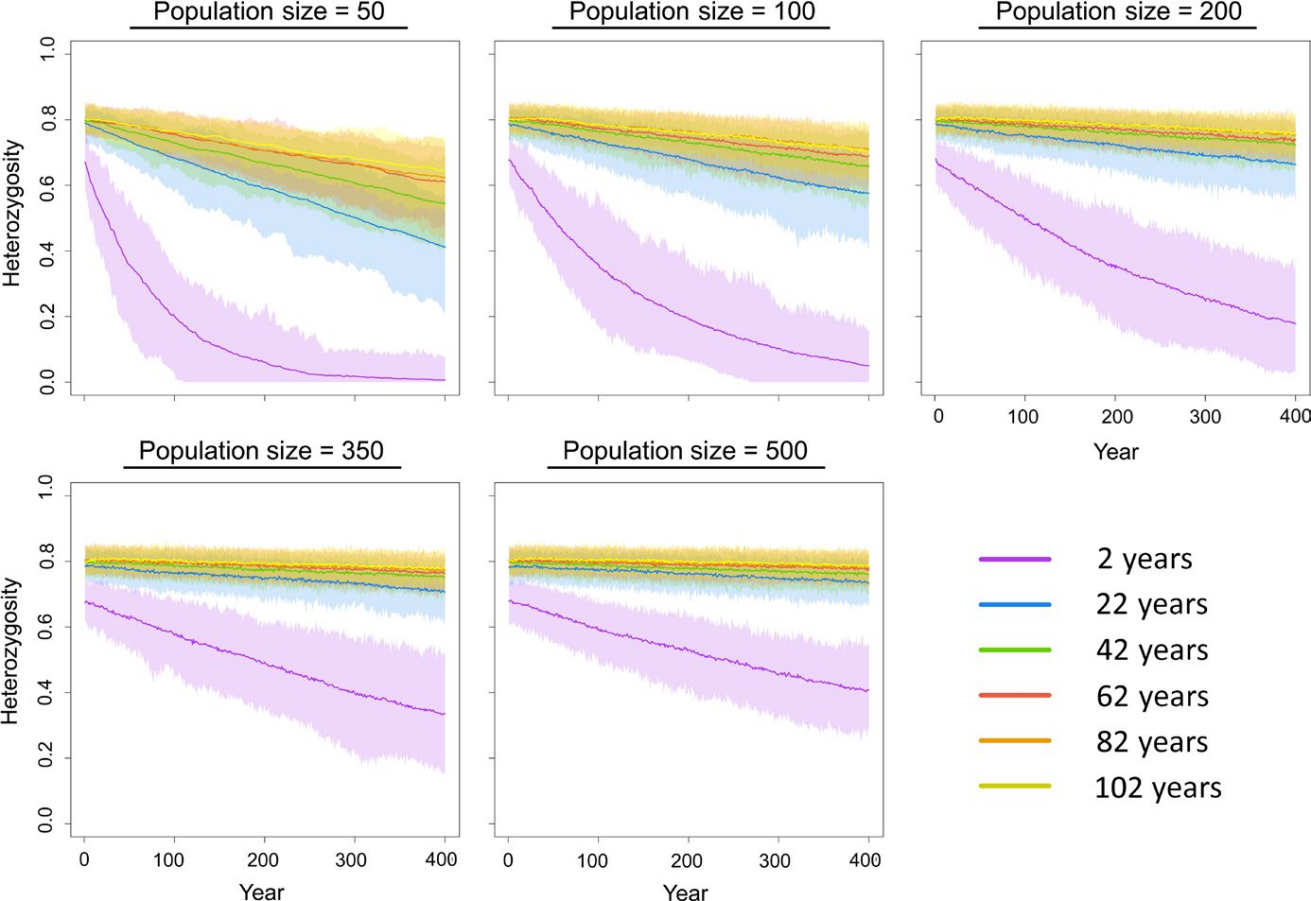
Average heterozygosity in simulated *Quadrula quadrula* populations of different *N*_c_ (50–500) and lifespans (2–102 years) over time

In all iterations and simulated population sizes, alleles were steadily lost from the population through time. Smaller populations tended to lose alleles and heterozygosity faster and were more prone to fixation at one or more loci compared to larger populations. Fixation of loci was more common in loci with fewer alleles or a greater disparity among initial allele frequencies. Similarly, heterozygosity of the upstream population fluctuated, but generally trended downward over time (Figure 3). This decrease in heterozygosity followed the loss of alleles or fixation.

**Figure 4:**
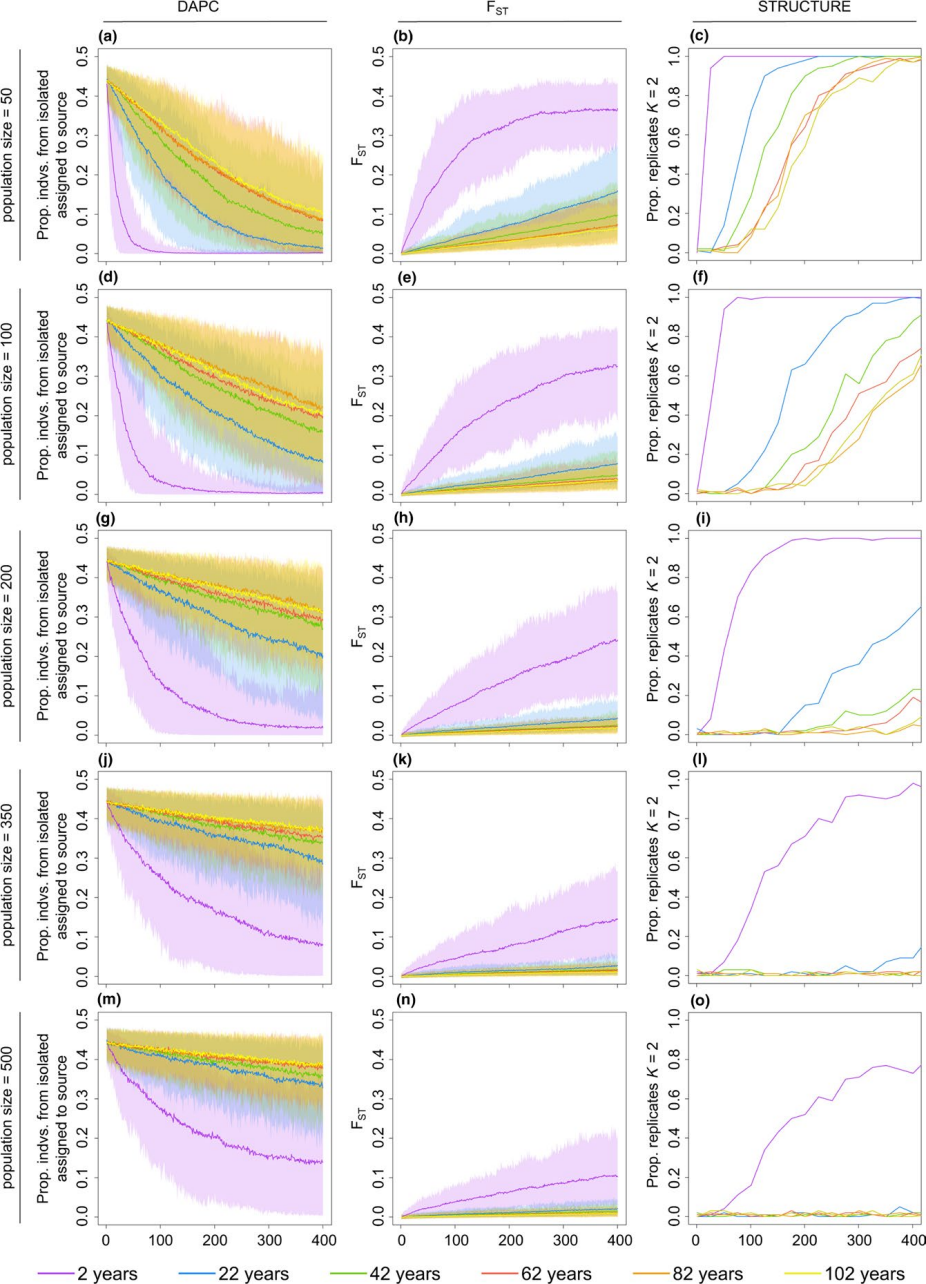
Results of discriminant analysis of principal components (DAPC), *F*_ST_, and STRUCTURE analyses across simulations of different *N*_c_ (50–500) and lifespans (2–102 years): The proportion of DAPC placement of individuals from the isolated upstream population into the source population over time (a, d, g, j, m), *F*_ST_ values between the isolated and source populations over time (b, e, h, k, n), and the proportion of STRUCTURE analyses that support two distinct populations (c, f, i, l, o). All values are averaged across simulation iterations. Simulations of populations with a lifespan of 62 years (red line) represent *Quadrula quadrula* examined in this case study

When maximum lifespan was 62, as is expected for *Q. quadrula*, the likelihood of identifying two distinct *Q. quadrula* populations using STRUCTURE approximated a logistic curve (Figure 4). At a small population size (*N*_c_ = 50), upstream and downstream mussels could be discerned as individual, divergent populations with 58% accuracy after 175 years of complete isolation (nearly the age of the Dunnville Dam), and with 98% accuracy after 375 years (Figure 4c). However, with *N*_c_ = 100, detection of genetic differentiation does not occur at that high a frequency, even after 400 years of isolation (Figure 4f). Over the 400 years of the simulation tested with analyses of genetic structure, structure was not detectable at frequencies higher than 20% for *N*_c_ > 200 using STRUCTURE analyses. Similar results were observed in *F*_ST_ and DAPC analyses; after 400 years and for *N*_c_ = 50, average *F*_ST_ between the simulated isolated population and the larger, Lake Erie population increased linearly from 0 at year 0 to 0.073 at year 400. After 175 years of isolation, *F*_ST_ between the two simulated populations was 0.031 (Figure 4b). Likewise, under the same conditions, DAPC placed 23.7% of individuals from the isolated, upstream population into the larger, downstream population (76.3% correct placement) on average after 175 years, and 8.3% of individuals (92% correct placement) after 400 years (Figure 4a). At larger population sizes such as *N*_c_ = 100, however, *F*_ST_ values decrease to 0.019 at 175 years and 0.038 at 400 years (Figure 4e). Additionally, DAPC placement of isolated individuals into the downstream source population increases to 31.6% at 175 years and 19.9% at 400 years (Figure 4d). At *N*_c_ = 500, *F*_ST_ and DAPC values do not change dramatically from starting values over time, with *F*_ST_ at 0.006 at 181 years and 0.01 at 400 years, and DAPC at 41.2% at 175 years and 37.8% at 400 years.

Similar patterns were observed among population sizes at other simulated lifespans; the simulations exhibited the same pattern of increase in response lag as population size increased. Additionally, as longevity increased, more time was necessary to observe genetic differentiation across the dam (Figure 4). However, simulations of 2‐year‐maximum lifespan individuals exhibited rapid population genetic change compared to the other models, with the differences between them becoming more pronounced at larger population sizes. The rapid development of genetic differentiation between the two simulated populations for 2‐year‐maximum populations was visible in all three methods of genetic structure detection. The differences between simulations were also dampened as the maximum lifespan increased. This was particularly evident for models with maximum lifespans of 62, 82, and 102 years, which show similar patterns across all simulated population sizes. Differences among the longest simulated lifespans were minimal and may mark the presence of an asymptote, where the probability of survival past a certain age (as determined by the survival rate) is so small that individuals do not live to their maximum age.

## 4 DISCUSSION

STRUCTURE analysis of the empirical data from *Q. quadrula* above and below the Dunnville Dam revealed no genetic structure, identifying only a single genetic population. However, DAPC placed less than 10% of individuals from the upstream population downstream, indicating the presence of some structure between the populations. The *F*_ST_ value between the populations upstream and downstream of the Dunnville Dam of 0.047 is high, but *F*_ST_ data among *Q. quadrula* populations in Lake Erie tributaries reached values as high as 0.197, 0.265, and 0.316, yet were still not determined to be separate populations in STRUCTURE analyses (Hoffman, 2016; Paterson et al., 2015). The *F*_ST_ value between the populations separated by the Dunnville Dam is low in comparison. Collectively, these results are not particularly conclusive as to whether the Dunnville Dam is a barrier to gene flow for the species. This result was surprising; given the qualities of the dam and the likely host fish used by *Q. quadrula*, clear and consistent structure was expected. The Dunnville Dam is known to inhibit the upstream dispersal of walleye (*Sander vitreus*; MacDougall et al., 2007), and thus likely impacts other fishes similarly. Other studies have found evidence of low‐head dams acting as barriers to channel catfish dispersal (Butler & Wahl, 2011; Welker, 1967), and so a similar effect from the Dunnville Dam was expected. Although a denil‐style fishway was installed in 1994 to alleviate the barrier on walleye, the fishway was ineffective at facilitating dispersal of the target species (Bunt, Cooke, & McKinley, 2000). As such, it is unlikely that channel catfish make use of this fishway. However, the lack of genetic structure resulting from the dam does not signify the absence of major ecological and habitat effects on the aquatic organisms in the river (Nilsson & Berggren, 2000).

We designed and implemented a forward time model in order to better interpret a lack of population structure across a dispersal barrier. Across all tested population sizes, and with all three analyses of genetic structure, genetic assessment of the simulated populations was more likely to identify two distinct populations with increasing time in isolation. This result fits population genetic theory; noninfinite populations no longer experiencing gene flow will drift apart genetically with time (Wright, 1966). When isolated populations had 50 individuals (and lifespan was equal to 62), distinct upstream and downstream populations could be identified by STRUCTURE 58% of the time after 175 years in isolation, close to the 181 years that the Dunnville Dam has been in existence. Thus, if the dam acted as an upstream barrier to gene flow, and the *Q. quadrula* population above the dam was approximately 50 individuals, then STRUCTURE analysis would not have been a reliable method of identifying structure between the upstream and downstream sites after 181 years of fragmentation. *F*_ST_ and DAPC analyses of 181 years of simulated fragmentation also showed little population differentiation at small population sizes, particularly when lifespan was >22 years. Furthermore, the range in outcomes across replicates increased as population size decreased, likely due to the randomness associated with genetic drift (Figure 4). Although the precise size is unknown, the isolated upstream population is unlikely to be as small as 50 individuals; *Q. quadrula* in the lower Grand River (source population location) is estimated to number in the millions (Committee on the Status of Endangered Wildlife in Canada (COSEWIC), 2006) and approximately 35 km of occupied river exist between the Dunnville Dam and the next upstream dam, the Caledonia Dam (MacDougall et al., 2007) to support a robust, even if isolated, population. In the Sydenham River (a tributary of Lake St. Clair in the Laurentian Great Lakes), the resident northern riffleshell (*Epioblasma torulosa rangiana*) census population size was estimated to number between 10,000 and 30,000 individuals (Zanatta & Murphy, 2007), and it is likely that *Q. quadrula* follow similar trends. As we found in the simulation, the likelihood of observing population divergence within a set time frame decreases with increasing population size. Thus, the combination of our inability to detect divergence at a small, simulated population size and a high probability of a large actual population size means that identifying genetic divergence between Grand River *Q. quadrula* is unlikely. This simulation then casts doubt on our inability to detect divergence in the empirical data.

Our model simulated a situation in which the *Q. quadrula* population was split by the dam, eliminating upstream and downstream gene flow, but downstream gene flow likely occurs in our case study; the Dunnville Dam, a low‐head dam, is probably not an impassable barrier to fishes (with encysted glochidia) traveling down river, nor the sperm of unionids (Ferguson, Blum, Raymer, Eackles, & Krane, 2013), resulting in unidirectional gene flow. This gene flow would slow the development of structure among fragmented populations and increase the time to detectable divergence. However, the downstream *Q. quadrula* population, encompassing Lake Erie and many of its tributaries (Hoffman, 2016), is so large that the influx of immigrants from the smaller, upstream population is unlikely to dramatically influence the divergence time between the two populations.

The results of this case study illustrate the importance of understanding and considering biology and life history characteristics of the organisms studied in addition to population genetic data. While population genetic analyses can be useful and informative tools for conservation biologists and managers, these data inherently require time to show changes in patterns of dispersal and gene flow. Organisms with long lifespans and generation times require more time to show genetic response to ecological changes (Willoughby, Sundaram, Lewis, & Swanson, 2013), and large population sizes can reduce response time even further (Marsack & Swanson, 2009). Thus, similar to difficulties found in detecting bottlenecks (Davy & Murphy, 2014), population genetic analyses may fail to reveal the complete history of fragmentation or isolation, even in situations where clear and obvious barriers to dispersal and gene flow exist.

In the case of *Q. quadrula* in the Grand River, one might be tempted to conclude that the dam is not a barrier to gene flow without the evidence brought forward by the simulations in this study. However, the model revealed that even under a situation of complete isolation, insufficient time has passed to detect isolation resulting from genetic drift. Indeed, the simulation results indicate that, for species with large populations, it would take longer to observe genetic structure in isolated *Q. quadrula* (and most other unionid species) than the vast majority of American dams have existed (National Inventory of Dams (NID), 2015). Relative to population genetic concerns alone, these two conclusions warrant different responses by conservation managers; if the dam is not a barrier to gene flow for the population, there is no need to remove the dam, at least relative to *Q. quadrula* and other species with similarly long generation times, longevity, and large population size. However, if the populations are lagging in response to the isolation, removing the barrier can prevent the population from experiencing the effects of fragmentation and drift in the future.

Results of the simulations across a spectrum of maximum lifespans illustrate the impact of lifespan specifically on this divergence time. The 2‐year lifespan model, representing short‐lived species, underwent genetic differentiation considerably more rapidly than in models for more long‐lived species (22–102 years). This pronounced shift was particularly noticeable in the heterozygosity (Figure 3) and *F*_ST_ estimation (Figure 4); heterozygosity decreased and *F*_ST_ increased nonlinearly, whereas the change over time was linear for other lifespan models. This is due to exponentially greater generation turnover in the short‐lived population; if generation time is assumed to equal the maximum lifespan exhibited by a species, a species with a 2‐year‐long lifespan will turn over 11 times within one generation of a species with a 22‐year lifespan. However, a species with a 22‐year lifespan will only experience roughly two generations within one generation of a species with a 42‐year lifespan. As a result, the time it takes, in years, for a population to respond genetically to fragmentation and isolation increases dramatically as with increasing lifespan.

The results of our simulation illustrate the potential for error when species life history characteristics are not considered in conjunction with population genetics when assessing the impacts of fragmentation. Inherent delays in responses to connectivity changes, particularly in large populations and long‐lived species, make analyses of population structure unlikely be informative at timescales meaningful to conservation management. Additionally, the genetic consequences of intensive fragmentation, such as genetic divergence between populations and inbreeding within them, can be overshadowed by the more immediate population demographic and ecological effects, such as interference in migration, spawning or mating behavior (Liermann, Nilsson, Robertson, & Ng, 2012). As observed in this study, depending on the lifespan and generation time of the species, the negative demographic and ecological impacts from fragmentation need to be addressed sometimes centuries before the genetic impacts will become relevant. As such, we suggest that long‐term impacts on genetic structure alone should not be an argument for or against the removal of a barrier, save for special cases of short‐lived species or species existing at small *N*_e_. Rather, the negative effects of barriers on system functions, including discharge and flow regimes (Dynesius & Nilsson, 1994; Graf, 1999), dispersal (Pess, McHenry, Beechie, & Davies, 2008; Watters, 1996), and community assemblage structure (Dean et al., 2002; Helms, Werneke, Gangloff, Hartfield, & Feminella, 2011) should be favored as grounds for remediation or removal.

Recognizing when population genetic data does not reveal the current state of connectivity among populations, and when it is necessary to rely on alternative data, is critical to avoiding incorrect conclusions and recommendations for management of species or populations of conservation concern. As such, predictive models that anticipate when fragmentation and isolation are likely to be detected, like the case study described here, are useful tools for conservation managers. These models only require estimates of basic demographic information (population size, lifespan, age of reproductive maturity, and annual survival rate) and have the potential to assess the reliability of structure analyses and lend additional support to the conclusions and recommendations of managers. Furthermore, predictive models can be easily modified to permit evaluation of situation‐specific variables, such as migration rate between the source and isolated populations. Employing such a model with a simulated dataset prior to conducting population genetic and structure analyses can reveal the probability of genetic structure forming within the time frame of interest, given the typical lifespan of the species and the size of the population. In this way, our model can assist in research planning and strategizing with regard to the allocation of precious research funds.

## CONFLICT OF INTEREST

None declared.

## AUTHOR CONTRIBUTIONS

Jordan R. Hoffman: Jointly conceived research plan, performed case‐study data collection and population genetic analysis, wrote the initial model, analyzed data, wrote the manuscript; Janna R. Willoughby: Optimized model, ran simulations on dedicated server, collected and organized data, advised throughout the project; Bradley J. Swanson: Jointly conceived research plan, assisted in model design, advised throughout the project; Kevin L. Pangle: Assisted in model design and troubleshooting, advised throughout the project; David T. Zanatta: Assisted and advised on case‐study data collection and population genetic analysis, advised throughout the project.

## DATA ACCESSIBILITY

IsoDrift model code deposited on GitHub: https://github.com/jwillou/Isolation-Drift.
